# Targeting succinate metabolism to decrease brain injury upon mechanical thrombectomy treatment of ischemic stroke

**DOI:** 10.1016/j.redox.2023.102600

**Published:** 2023-01-02

**Authors:** Amin Mottahedin, Hiran A. Prag, Andreas Dannhorn, Richard Mair, Christina Schmidt, Ming Yang, Annabel Sorby-Adams, Jordan J. Lee, Nils Burger, Duvaraka Kulaveerasingam, Margaret M. Huang, Stefano Pluchino, Luca Peruzzotti-Jametti, Richard Goodwin, Christian Frezza, Michael P. Murphy, Thomas Krieg

**Affiliations:** a MRC Mitochondrial Biology Unit, University of Cambridge, The Keith Peters Building, Cambridge Biomedical Campus, Cambridge, UK; b Department of Physiology, Institute of Neuroscience and Physiology, University of Gothenburg, Gothenburg, Sweden; c Department of Medicine, University of Cambridge, Cambridge University Hospitals, Cambridge, UK; d Imaging and Data Analytics, Clinical Pharmacology and Safety Sciences, R & D, AstraZeneca, Cambridge, UK; e Division of Neurosurgery, Department of Clinical Neurosciences, Cambridge University Hospitals, Cambridge, UK; f CECAD Research Center, Faculty of Medicine, University Hospital Cologne, Cologne, Germany; g Department of Clinical Neurosciences and NIHR Biomedical Research Centre, University of Cambridge, UK

## Abstract

Current treatments for acute ischemic stroke aim to reinstate a normal perfusion in the ischemic territory but can also cause significant ischemia-reperfusion (IR) injury. Previous data in experimental models of stroke show that ischemia leads to the accumulation of succinate, and, upon reperfusion, the accumulated succinate is rapidly oxidized by succinate dehydrogenase (SDH) to drive superoxide production at mitochondrial complex I. Despite this process initiating IR injury and causing further tissue damage, the potential of targeting succinate metabolism to minimize IR injury remains unexplored. Using both quantitative and untargeted high-resolution metabolomics, we show a time-dependent accumulation of succinate in both human and mouse brain exposed to ischemia *ex vivo*. In a mouse model of ischemic stroke/mechanical thrombectomy mass spectrometry imaging (MSI) shows that succinate accumulation is confined to the ischemic region, and that the accumulated succinate is rapidly oxidized upon reperfusion. Targeting succinate oxidation by systemic infusion of the SDH inhibitor malonate upon reperfusion leads to a dose-dependent decrease in acute brain injury. Together these findings support targeting succinate metabolism upon reperfusion to decrease IR injury as a valuable adjunct to mechanical thrombectomy in ischemic stroke.

## Graphical abstract

**Figure:**
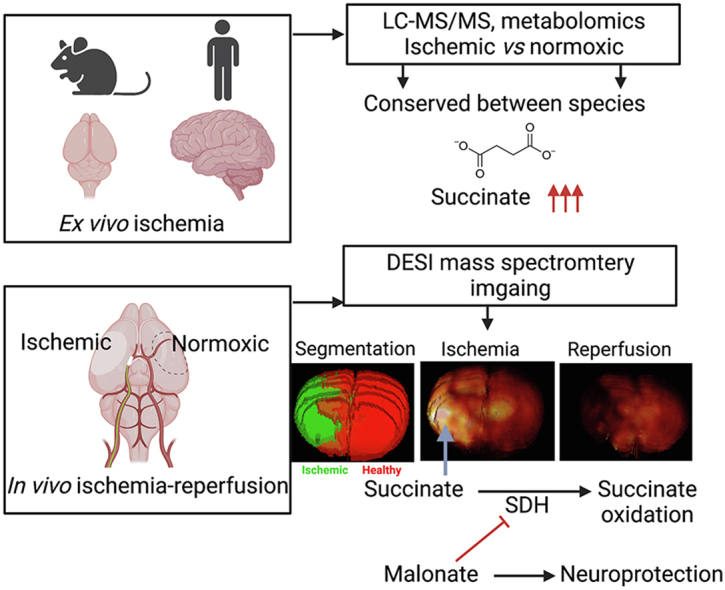

## 1 Introduction

Stroke is a leading cause of death and disability worldwide with ischemic stroke being the most prevalent subtype [1]. Current treatments for ischemic stroke are limited to restoring circulation by removal of the occluding clot, which has historically been achieved using intravenous proteases such as tissue plasminogen activator (tPA). Nowadays, removal of the occluding clot is increasingly achieved by mechanical thrombectomy, either alone or in combination with tPA, which has clear advantages, particularly in cases of large vessel occlusion, as it immediately leads to a targeted restoration of blood flow [2]. Moreover, mechanical thrombectomy has shown efficacy up to 24 h post-stroke, markedly extending the narrow time window for treatment [[3], [4], [5], [6], [7]]. However, while rapid restoration of blood flow following ischemic stroke by mechanical thrombectomy is essential to rescue ischemic tissue, the IR injury associated with reperfusion can also contribute to pathology, and as a result, the disability-adjusted loss of life-years following stroke remain stubbornly high [1]. The cerebral IR injury that occurs following mechanical thrombectomy is thought to arise from a burst of superoxide production upon reperfusion of the ischemic tissue [8]. Superoxide generation initiates a cascade of oxidative damage that causes acute tissue injury and leads to further tissue inflammation and gliosis [9]. As the exact time of the reperfusion of the ischemic tissue after mechanical thrombectomy is known, there exists an opportunity to combine revascularization with an adjunct therapy to decrease the associated acute IR injury [8].

Recently, in our work on cardiac IR injury, we showed that the mitochondrial metabolite succinate accumulates dramatically within tissues during ischemia and is then rapidly oxidized by succinate dehydrogenase (SDH) at reperfusion, driving superoxide production at mitochondrial complex I via reverse electron transport (RET), which initiates IR injury [[10], [11], [12], [13], [14]]. Importantly, blocking succinate oxidation at SDH with cell-permeable pro-drug esters of its competitive inhibitor, malonate, ameliorates myocardial infarction [10,15]. Furthermore, we recently showed that malonate itself, administered as disodium malonate (DSM), is selectively taken up into ischemic tissues by monocarboxylate carriers (MCT) due to the lowered pH and high local lactate concentration [15]. Consequently, DSM is highly effective at decreasing cardiac IR injury *in vivo* when administered at the clinically-relevant point of reperfusion [15]. In addition, preloading the brains of spontaneously hypertensive rats with malonate by administering cell-permeable malonate esters prior to ischemia inhibited succinate accumulation and was protective against IR injury in an ischemic stroke model [10]. Together, these data support succinate accumulation during the ischemic phase of stroke followed by its rapid oxidation upon reperfusion when the occluded blood vessel is unblocked by mechanical thrombectomy. This model suggests that administering malonate at the same time as mechanical thrombectomy may have potential as an adjunct therapy.

To address this question, we used both quantitative targeted, and high-resolution untargeted metabolomics to compare the metabolic responses to ischemia in human and mouse brain. In parallel, we used mass spectrometry imaging (MSI) to visualize changes in succinate levels in a mouse model of stroke during both ischemia and following reperfusion in a way that mimics mechanical thrombectomy. Finally, we assessed whether administration of malonate as DSM upon reperfusion following ischemia was protective against acute brain injury. Together, our findings support the potential of malonate infusion during mechanical thrombectomy as an adjunct therapy for the treatment of ischemic stroke.

## 2 Results

**Fig. 1:**
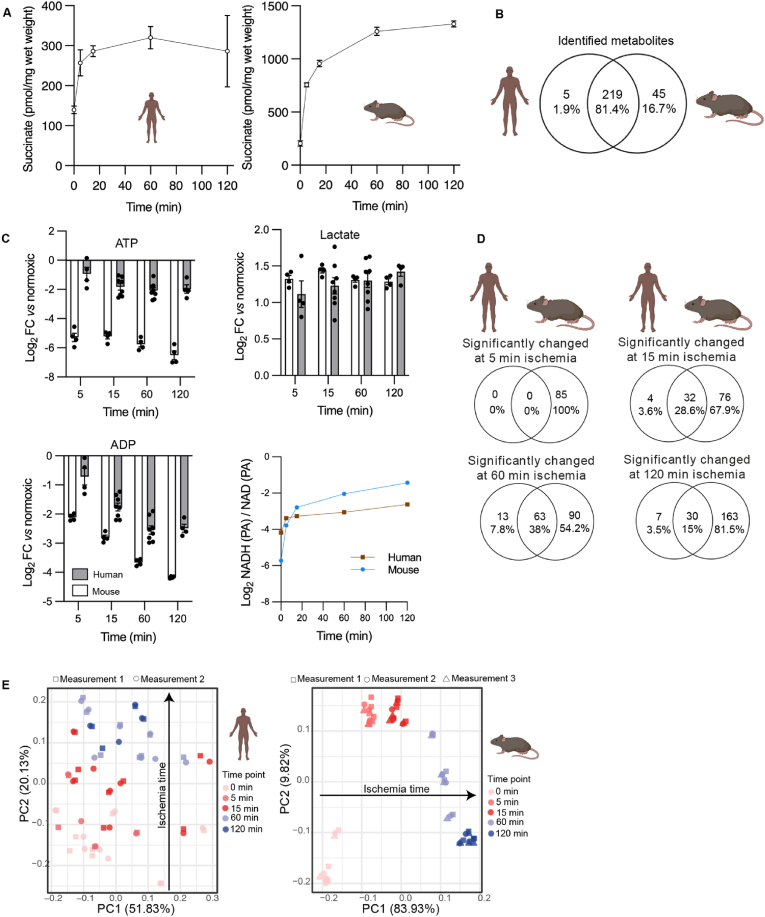
**Time-resolved metabolomics of human and mouse brain during normoxia and in response to warm ischemia *ex vivo*. (A)** Succinate accumulation quantified by LC-MS/MS in mouse and human brain. Data are mean ± SEM. n = 4–8 human, n = 4 mice. **(B)** Venn diagram of the distinct metabolites detected by LC-MS in normoxic mouse and human brain. Metabolites are listed in Supplemental Tables 2 and 3**(C)** Changes in energy metabolism during warm ischemia *ex vivo* for human and mouse brain samples determined by LC-MS. Data are from Supplemental Tables 2 and 3 ATP, ADP and lactate are shown as Log_2_ Fold Change (FC) of metabolite intensity measured as peak areas (PA) for that sample. For the ratio of NADH and NAD PAs, the data show the ratio in these values determined for each sample. Note that the ratio of NADH and NAD^+^ PAs is related to but is not the same as the NADH/NAD^+^ molar ratio. Data are mean ± SEM. n = 4–8 human, n = 4 mice. **(D)** Venn diagrams showing the distinct metabolites detected in the mouse and human brain samples over different durations of *ex vivo* warm ischemia. Metabolites are listed in Supplemental Tables 2 and 3**(E)** PCA analysis of the changes in levels of metabolites during *ex vivo* warm ischemia for mouse and human brain samples. Metabolites are listed in Supplemental Tables 2 and 3

*Succinate accumulates in human and mouse brains during ischemia*. We compared the metabolite content of whole mouse brains with small amounts of healthy human brain tissue, which was collected during neurosurgical tumor resection (Supplemental Table 1). While human samples varied in brain location, sex and age of donor, they nevertheless provided a valuable resource to compare the metabolic profile of mouse and human brain tissue during *ex vivo* warm ischemia, which was designed to mimic the metabolic changes that occur in the brain during ischemic stroke (Fig. 1). We minimized the time from dissection to freezing in theatre to less than a minute, to obtain samples as close to normoxic conditions as possible and compared these to rapidly isolated and frozen whole mouse brains. We found that succinate showed rapid increases over time upon ischemia in both mouse and human (Fig. 1A).

### 2.1 Time-resolved metabolic profiling of human and mouse brain during ischemia

We next analyzed the normoxic brain tissue samples by untargeted high-resolution liquid chromatography-tandem mass spectrometry (LC-MS) to expand our metabolomic profiling. We identified 264 and 224 metabolites in the normoxic mouse and human brains, respectively (Supplementary Tables 2 and 3). Of these, 81% were identified in both species (Fig. 1B). The changes in these metabolites over time were also assessed during warm ischemia in human (Supplementary Table 2) and mouse (Supplementary Table 3) brain tissue. Ischemia led to similar and rapid decreases in ATP (3.2-fold reduction in human by 15 min and 36.3-fold reduction in mouse) and ADP (3.2-fold reduction in human by 15 min and 6.9-fold reduction in mouse) levels in both species compared to normoxia (Fig. 1C). There were also increases in lactate (2.4-fold increase in human by 15 min and 2.7-fold increase in mouse, compared to normoxia) and in the peak area ratios for NADH and NAD^+^ compared to normoxia in both species (Fig. 1C). The magnitude of these changes in metabolite levels upon warm ischemia compared to normoxia suggest that the human brain samples were frozen fast enough to avoid a major induction of ischemic metabolism. The broad similarity in the statistically significant changes in metabolites over time between mouse and human is further illustrated in the Venn diagrams, showing that most of the metabolites altered in the human brain during warm ischemia, were also changed during warm ischemia in the mouse brain (Fig. 1D). Principal component analysis (PCA) showed a clear pattern of metabolite changes during warm ischemia in both human and mouse brain, while this is more pronounced in mouse where ischemia duration accounts for 83.9% of metabolite changes (Fig. 1E). This pattern was not as pronounced in the human samples, presumably in part due to the variation in patients’ age, sex and health status (Supplemental Table 1), or to the sampling of only small sections of the human brain, due to the constraints of surgery (Fig. 1D, Supplemental Table 1).

### 2.2 Quantification of metabolic changes in the mouse and human brain during warm ischemia

**Fig. 2:**
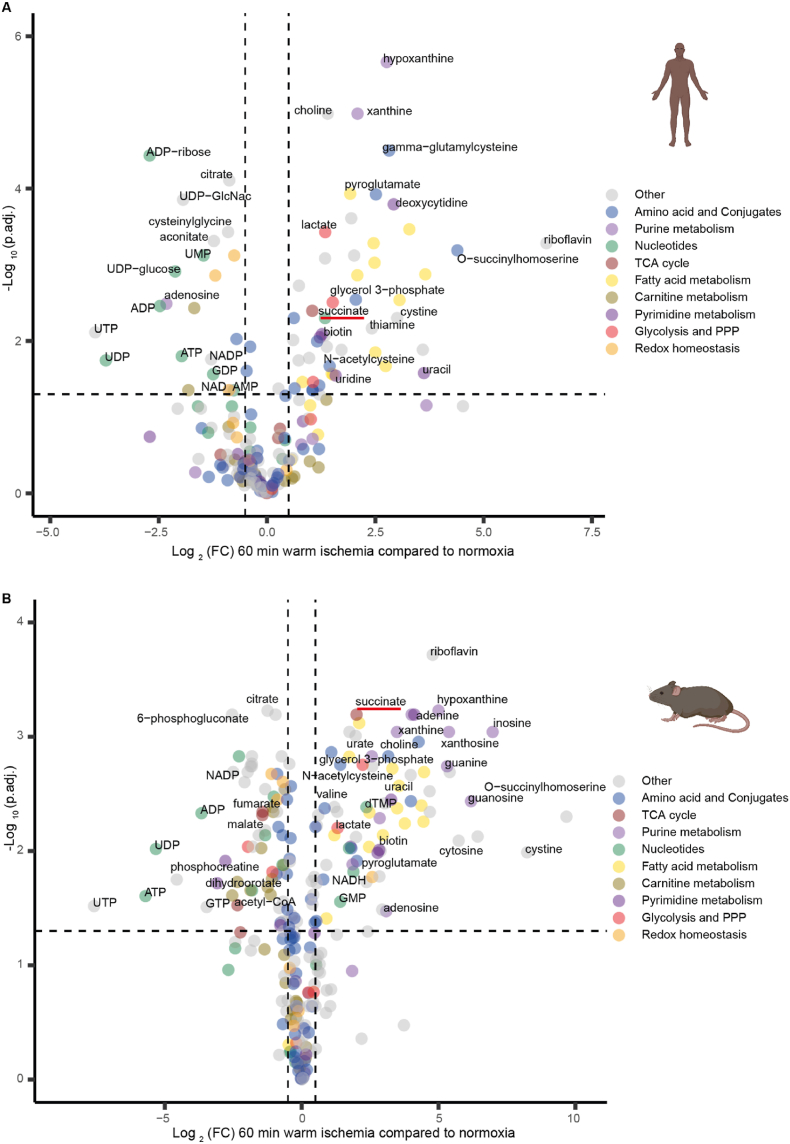
**Volcano plots showing changes in metabolite levels following 60 min *ex vivo* warm ischemia compared to normoxia**. Human **(A)** and mouse **(B)** brain tissue data are shown. The volcano plots show -Log_10_(adjusted p value (p.adj.) calculated by Benjamini-Hochberg procedure), plotted against Log_2_(fold change (FC)), where fold change is that comparing the change in abundance of metabolites after 60 min warm ischemia versus normoxia. Metabolite abundances are given in Supplemental Tables 2 and 3 and Log_2_(FC) and -Log_10_(p.adj.) were calculated from these data. Each dot represents the mean of 8 replicates for human and of 4 replicates for mouse. Metabolites from different metabolic pathways are color-coded and key metabolites and/or those that change to the greatest extent are labeled. (For interpretation of the references to color in this figure legend, the reader is referred to the Web version of this article.)

To facilitate the comparison of metabolic changes relative to normoxia during ischemia in mouse and human tissues, we represented these data as volcano plots. Warm ischemia of 60 min led to the largest number of metabolites showing differential abundance compared to normoxia, in both human and mouse brains (Fig. 1D). Therefore, we interrogated these data further by plotting them as volcano plots for human (Fig. 2A) and mouse (Fig. 2B) brain samples, with color coding for metabolic pathways of interest. These plots showed considerable similarity between human and mouse brain, as did those after other durations of warm ischemia (Supplemental Figs. 1–3). Similar to data in Fig. 1A, we observed a 2-fold increase in succinate level in human brain and 3.5-fold increase in mouse brain after 60 min of ischemia. Some other notable changes that occurred in both mouse and human brain following ischemia were an increase in products of purine degradation such as hypoxanthine (6.7-fold increase in human, 31.9-fold increase in mouse) and xanthine (4.2-fold increase in human, 11.1-fold increase in mouse). There was also a decrease in several acylcarnitines over time, and pyroglutamate (5.7-fold increase in human and 4-fold increase in mouse) was among the most significantly accumulated metabolites in both species. There were also similar changes in unsaturated long-chain fatty acids (e.g. c20:4, c22:4, c22:5), which may be indicative of increased lipolysis of phospholipids. Adenosine increased during ischemia in the mouse brain (8.4-fold increase, p = 0.033) but decreased in the human brain (5-fold reduction, p = 0.003). Moreover, inosine, which is formed by deamination of adenosine, remained stable in the ischemic human brain, while its level increased during ischemia in the mouse brain (Supplemental Tables 2 and 3). Five metabolites (*N*-acetylputrescine, ophthalmate, raffinose, dihydrobiopterin, sarcosine) were detected in human but were below the limit of detection in mouse brain, although the levels of these metabolites did not change in response to ischemia (Supplemental Table 2). Thus, the metabolic changes in human and mouse brain during prolonged ischemia are broadly similar, and, in particular, we see an accumulation of succinate, corroborating its potential as a therapeutic target.

### 2.3 Mouse model of stroke and mechanical thrombectomy

**Fig. 3:**
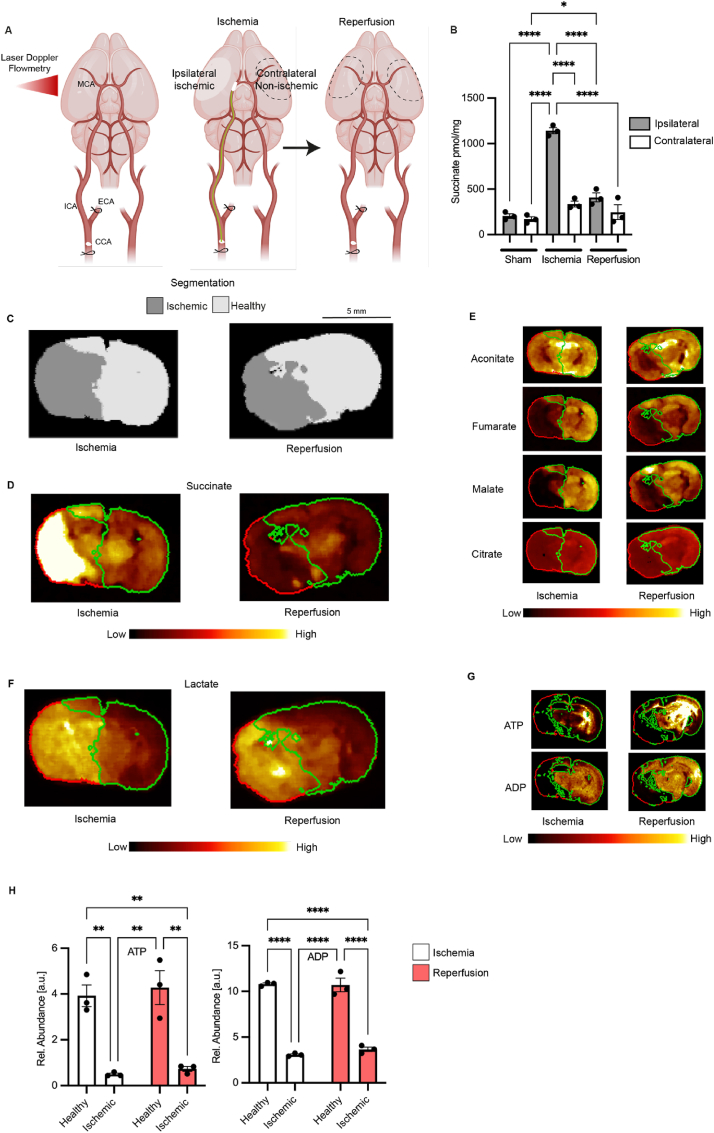
**Metabolite distribution in a mouse stroke model of tMCAO followed by reperfusion. (A)** Schematics of the tMCAO stroke model in mice. A standard silicon-tip suture was used to occlude the MCA and cerebral flow was monitored by Laser Doppler Flowmetry to confirm the ischemia. MCA, ECA, ICA and CCA are middle, external, internal and common carotid arteries respectively **(B)** LC-MS/MS measurement of succinate in the brain of mice subjected to tMCAO (45 min ischemia or 45 ischemia and 5 min reperfusion) or sham operation. Data are presented as mean ± SEM, n = 3. Comparisons between groups was performed using one-way ANOVA and Tukey's test. Cont. = contralateral, Ipsi. = ipsilateral (**C**) Segmentation of the brain after 45 min ischemia and after 45 min ischemia and 5 min reperfusion assessed shows healthy (light grey) and ischemic (dark grey) tissue. (**D**) Distribution of succinate in the brain after 45 min ischemia and after 45 min ischemia and 5 min reperfusion. Segmentation is done as in C and is indicated by red and green outlines for healthy (green) and ischemic (red) tissue. (**E**) Distribution of TCA metabolites. (**F**) Distribution of lactate. (**G**) Distribution of ATP and ADP. (**H**) Quantification of ATP and ADP. Comparisons between groups was performed using two-way ANOVA and Sidak's test, n = 3. (For interpretation of the references to color in this figure legend, the reader is referred to the Web version of this article.)

The above analysis indicated that succinate accumulates during global warm ischemia in both human and mouse brain tissue *ex vivo*. However, only a localized area of the brain is affected during ischemic stroke. Therefore, assessing the distribution of succinate within the brain during ischemic stroke *in vivo* is key to understanding its potential role in pathology. We used a mouse model of transient Middle Cerebral Artery Occlusion (tMCAO) [16] (Fig. 3A), whereby an intraluminal filament was inserted into the MCA, left *in situ* for 45 min and then removed, to model mechanical thrombectomy. This approach achieves fast reperfusion, which closely replicates the rapid reperfusion of ischemic tissue upon restoration of blood flow by mechanical thrombectomy in patients [16].

### 2.4 Distribution of succinate during ischemia and after reperfusion in a mouse model of stroke

To assess the potential contribution of succinate metabolism to infarct formation and evolution, we measured its accumulation by LC-MS/MS during ischemia in the *ipsilateral* and *contralateral* hemispheres of the mouse brain and compared these with sham operated mice that had not been exposed to ischemia (Fig. 3B). During ischemia succinate accumulated 4-5-fold in the *ipsilateral*, but not in the *contralateral* hemisphere. After reperfusion for 5 min the level of succinate in the *ipsilateral* brain hemisphere had returned to that of normoxic tissue.

### 2.5 Assessment of succinate distribution during ischemia and after reperfusion in stroke by mass spectrometry imaging (MSI)

For the LC-MS/MS quantification of succinate in the *ipsilateral* hemisphere the area at risk was identified subjectively, based on knowledge of infarct location in previous experiments. However, this tissue may include both healthy and infarcted brain regions. To better determine the distribution and relative quantification of a range of metabolites over both brain hemispheres we applied Desorption Electro Spray Ionization-imaging mass spectrometry (DESI-MSI) to brain slices. To ensure metabolic integrity of the samples, the whole brains were rapidly removed after ischemia, or after ischemia-reperfusion, and rapidly frozen in liquid-nitrogen cooled isopentane. The frozen brains were then cryo-sectioned into 10 μm slices. To cover the spatial arrangement of metabolic changes across the entire brain, the MSI data from these tissue slices were collected in 1 mm increments. These tissue sections were subjected to untargeted DESI-MSI analysis to assess a wide range of metabolites, including structural lipids, free fatty acids, tricarboxylic acid (TCA) cycle and glycolytic intermediates, and other metabolites of potential interest. Assessment of the metabolite distribution coupled to image segmentation by PCA and unsupervised bisecting k-means clustering, allowed us to identify the non-ischemic and ischemic regions of the mouse brain subjected to tMCAO (Fig. 3C). Further analysis after ischemia followed by 5 min reperfusion identified the area of tissue affected by IR injury (Fig. 3C). Analysis of the same brain slices using MSI based on an alternative ionization modality, matrix-assisted laser desorption/ionization (MALDI)-MSI, allowed us to extend the investigation to include high-energy phosphates such as ATP and ADP, as well and AMP, which showed a similar localization for the ischemic lesion, as well as that after ischemia followed by 5 min reperfusion which we identified as the area of tissue affected by IR injury (Supplemental Fig. 4).

The segmentation of the DESI-MSI data within the brain slices enabled us to demarcate the ischemic region of the brain and also the tissue damaged following reperfusion. Overlaying this pattern (red line) onto the brain enabled us to extract statistical information for the resolved tissue segments and thus quantify the relative change in abundance from the metabolite distribution maps. This analysis showed that succinate was accumulated in the ischemic lesion site within the *ipsilateral* brain hemisphere (Fig. 3D, Supplemental Fig. 5). Importantly, succinate that accumulated in this region during ischemia was rapidly lost upon reperfusion (Fig. 3D, Supplemental Fig. 5). Similar DESI-MSI analysis enabled us to map changes in a range of other metabolites onto the lesions formed during ischemia as well as those generated upon subsequent reperfusion. In contrast to succinate, other TCA cycle metabolites were depleted during ischemia (Fig. 3E, Supplemental Fig. 5), consistent with the selective accumulation of succinate during ischemia [10,17]. Lactate did accumulate and interestingly its levels remained elevated after reperfusion (Fig. 3F). ATP and ADP decreased dramatically in the ischemic lesion upon induction of ischemia and did not regenerate when measured again after 5 min of reperfusion (Fig. 3G and H) pointing toward an incomplete re-establishment of cellular function and energy metabolism in affected brain areas upon reperfusion. Changes in abundance of other key metabolites in purine nucleotide breakdown and glycolysis pathways in the ischemic lesion mirror that of *ex vivo* ischemia in mouse brain (Supplemental Fig. 5). These findings of succinate accumulation during ischemia followed by its rapid loss upon reperfusion are consistent with succinate oxidation at SDH during the treatment of ischemic stroke by mechanical thrombectomy.

### 2.6 In vivo administration of malonate upon reperfusion reduces acute brain injury in a mouse model of stroke

**Fig. 4:**
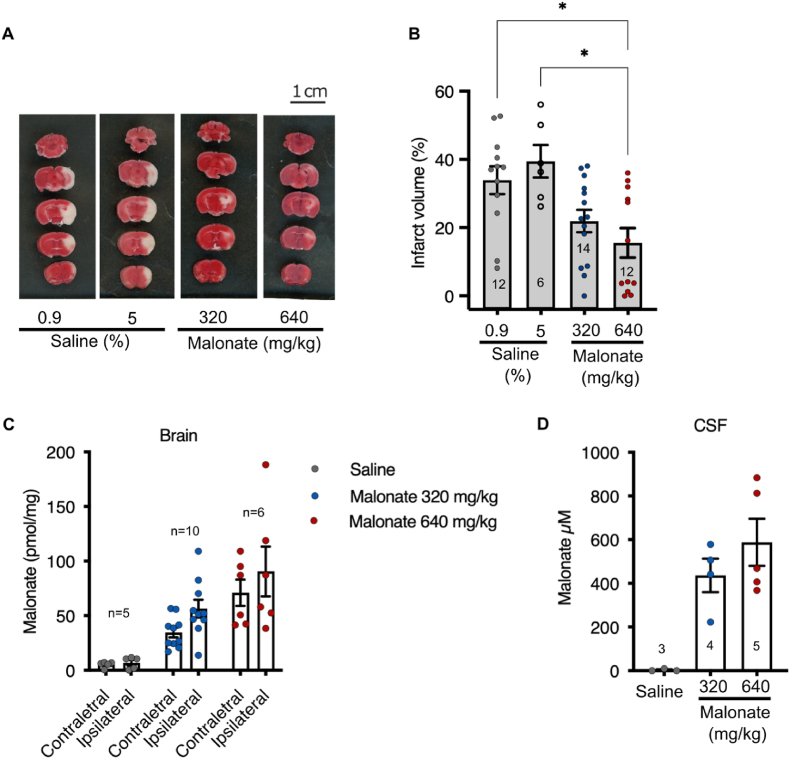
**Effect of malonate infusion in a mouse stroke model of tMCAO followed by reperfusion. (A)** Mice were exposed by tMCAO to 45 min of ischemia and either saline (0.9 or 5%) or disodium malonate (DSM) 320 or 640 mg/kg was given intravenously starting 10 min before reperfusion until 10 min after reperfusion. Two hours after reperfusion mice well culled and TTC staining was performed. Typical data are shown. **(B)** Quantification of infarcts size from experiments carried out as in (A). Number of mice per group are shown in the bars. Data are presented as mean ± SEM and *p < 0.05 by Kruskal Wallis and Dunn's test. **(C, D)** Mice were treated as described in (A) and 10 min after reperfusion the brain circulation was cleared of blood with saline by cardiac perfusion. Then contralateral and ipsilateral brain hemispheres (C) and CSF (D) were isolated and malonate content determined by LC-MS/MS. Number of mice per group are shown above or within the bars. Data are presented as mean ± SEM.

The above findings show that succinate accumulates substantially in the brain during ischemia and then is rapidly oxidized upon reperfusion. These results are consistent with mitochondrial superoxide production driven by succinate oxidation upon reperfusion contributing to brain injury following mechanical thrombectomy and suggest that inhibiting SDH upon reperfusion may offer protection against IR injury [[13], [14], [15]]. Therefore, we next administered malonate, as its disodium salt DSM, intravenously for 20 min, starting 10 min prior to removal of the intraluminal filament and continuing as the ischemic brain tissue was reperfused (Fig. 3A). Cerebral blood flow (CBF), assessed by laser Doppler flowmetry correlated with periods of ischemia and reperfusion, and blood flow was not affected by DSM treatment (Supplemental Fig. 6). Infusion of DSM significantly decreased acute brain injury, as measured by 2,3,5-triphenyltetrazolium chloride (TTC) staining and histology (Fig. 4A and B) and led to significant uptake of malonate into the brain tissue (Fig. 4C) and CSF (Fig. 4D). These findings suggest that the protection against acute IR injury afforded by malonate follows its uptake into brain tissue, indicating that malonate can pass the blood-brain and blood-CSF barriers under these conditions, and is consistent with its protection by inhibiting SDH.

## 3 Discussion

The current standard of care for ischemic stroke is to restore blood flow as rapidly as possible, which is increasingly being achieved by mechanical thrombectomy. Even so, this procedure is associated with considerable brain tissue injury due to the inevitable IR injury upon the reperfusion of ischemic tissue. Here we show that the metabolic changes occurring during ischemia in mouse and human brain samples have notable similarities. In particular, we found that the selective accumulation of the mitochondrial metabolite succinate occurred in both species. In a mouse model that mimics the treatment of ischemic stroke by mechanical thrombectomy, we found that the succinate that accumulated during ischemia was localized to the ischemic lesion. Furthermore, upon restoration of blood flow, succinate levels very rapidly returned to those of normoxic tissue. As succinate oxidation by SDH upon the reperfusion of ischemic tissue is a major driver of IR injury, we hypothesized that inhibiting this oxidation using the SDH inhibitor malonate might lower the brain damage. This was based on recent work by us and others, which established that infusion of malonate was protective against IR injury in the heart [10,13,14]. Herein we show that infusing malonate upon reperfusion of the ischemic brain protected against acute brain injury as well. Thus, malonate infusion may be a potential adjunct therapy to mechanical thrombectomy for the treatment of ischemic stroke. In addition to the potential therapeutic insights, we also report a high-resolution time-resolved metabolome of human and mouse brain in response to warm ischemia *ex vivo*. This may serve as a useful resource in translational stroke research.

Up to now, data on human brain metabolic response to ischemia have been limited to that obtained by proton [^1^H]-magnetic resonance spectroscopy (MRS) in stroke patients. Despite its clinical usefulness in visualizing the core stroke lesion by detecting metabolites such as N-acetylaspartate and lactate [18], MRS lacks the sensitivity to capture metabolic changes lower than millimolar [19]. Most importantly, we show that succinate accumulation is a conserved phenomenon in ischemia of human and mouse brain, despite some differences in changes in other TCA cycle metabolites between species in response to ischemia. Both human and mouse brain also showed a shift toward glycolysis as well as similar changes in fatty acid and carnitine metabolism in response to ischemia. One divergence between human and mouse was in purine metabolism. While the amounts of xanthine and hypoxanthine were both increased in ischemic human and mouse brain, adenosine increased in the ischemic mouse brain as shown previously [20,21], but was depleted in ischemic human brain. This may have clinical implications considering the reported vasodilatory and anti-excitotoxicity effects of adenosine and attempts to target its receptors in pre-clinical stroke trials [22].

Our study inevitably has several limitations. Although, the brain samples obtained from cancer patients were not part of the tumor, we cannot rule out the possibility that the metabolism of tissues in proximity to the tumor may be affected. Moreover, the *ex vivo* warm ischemia experiment may not capture all properties of the ischemic condition *in vivo*, such as mechanical forces caused by edema. Surface exposure to the ambient air in *ex vivo* experiment may also affect the extent of the anoxia. In addition, malonate treatment in the experimental stroke mouse model was intended to test the concept of targeting SDH at reperfusion to reduce infarct size during the acute phase of the pathology. Further experiments are needed to explore the long-term effects of this treatment on the lesion development, neurological outcome, markers of oxidative damage and potential toxicity on other organs.

In summary, this work sheds lights on the similarities of metabolic response of human and mouse brain to warm ischemia and shows that the changes in succinate metabolism are conserved during ischemia. This suggests the inhibition of succinate oxidation upon reperfusion as a potential translatable intervention for the treatment of ischemic stroke in combination with mechanical thrombectomy.

## 4 Methods

### 4.1 Human brain experiments

Human brain samples were obtained from 8 patients undergoing neurosurgery for removal of different types of brain tumors. Non-tumor normal brain tissue for surgical access was obtained from regions that were non-enhancing and not associated with Fluid-attenuated inversion recovery (FLAIR) signal abnormality on intra-operative neuro-navigation, co-registered with pre-operative MRI. The patients’ characteristics and anatomical locations of the samples are indicated in Supplemental Table 1. To avoid incomplete anoxia due to large surface/mass ratio and ambient air exposure, only samples >5 mg wet weight of brain tissue (excluding vasculature) were analyzed. Samples from one patient that did not meet this criterion were excluded. Immediately after obtaining the brain tissue, the sample was cut into several pieces (depending on the sample size) in an adjacent lab and one piece was immediately snap-frozen in liquid nitrogen (LN2) as the non-ischemic normoxic control (0 min). In cases were more than one piece was cut out by the surgeon, one whole piece was snap-frozen in the surgery theatre as the normoxic control. The individual times taken until the normoxic control samples were frozen are indicated in Supplemental Table 1 and for most samples this was within 1 min. To obtain ischemic tissue, the additional tissue pieces were incubated in a humidified Eppendorf tube at 37 °C for indicated time periods and then snap frozen in LN2. Frozen samples were stored in −80 °C freezer until the final analysis.

### 4.2 Mice

Male, adult C57BL6J mice were purchased from Charles River Laboratories and housed in a room on a 12h light/dark cycle with *ad libitum* access to food and water. Mice were acclimatized in our facility for a week before being used in the experiments. At the time of experiments mice were aged 8–12 weeks and weighed 25–32 g.

### 4.3 Mouse brain *ex vivo* ischemia model

Mice were sacrificed by cervical dislocation and the whole brain was either immediately snap-frozen in LN2 for control normoxic tissue or put in an incubator at 37 °C for indicated time periods to induce global ischemia. Samples were kept at −80 °C until subsequent analysis.

### 4.4 Transient middle cerebral artery occlusion (tMCAO) model of stroke

Mice were anesthetized by isoflurane (3% induction, 1.5–2% maintenance) delivered in 100% oxygen. A Doppler (PeriFlux System 5000, Perimed, Sweden) flowmetry probe was attached to the skull on the ipsilateral side to monitor and record the blood flow in the MCA territory. Next, the left common carotid artery (CCA) and external carotid artery were exposed and permanently ligated. After temporarily occluding the left internal carotid artery by a clamp, an incision was made on CCA and a 6-0 suture with a silicone-coated tip (602223PK10RE, Doccol, USA) was inserted. The clamp was removed, and the suture was inserted forward until a drop in blood flow was observed on Doppler confirming the MCAO and the start of ischemia. Animals that showed >70% drop in cerebral blood flow in relation to the baseline were included in the study. After an ischemia duration of 45 min the suture was removed, allowing the reperfusion for indicated periods of time. The mice were sacrificed by cervical dislocation and brains were collected for infarct measurement. For measurement of malonate some mice were killed with an overdose (∼350 mg/kg) of pentobarbital and perfused through the heart with ice-cold saline to clear the blood. Then, the cerebral spinal fluid (CSF) was collected from cisterna magna using a glass capillary and brain samples were collected and clamp-frozen in LN2.

### 4.5 Measurement of infarct volume

Mice underwent tMCAO surgery with 45 min of ischemia followed by 2 h reperfusion and were sacrificed by cervical dislocation. Brains were dissected out and immediately sliced at 2 mm thickness and stained in pre-warmed 2% TTC at 37 °C for 8 min to identify the infarct area. The slices were then fixed in 4% paraformaldehyde overnight at 4 °C and were imaged using a scanner. The healthy area (stained red) was measured in two hemispheres. The infarct volume was calculated as sum of (infarct area × slice thickness) and was presented as percentage of the healthy hemisphere volume [23]. When no infarct was detected, the percentage was indicated as 0.

### 4.6 Extraction of metabolites

Human and mouse brain samples were weighed on dry ice and placed in pre-chilled Precellys tube (CK28-R, Bertin Instruments, France). For human brain, the mean ± SD of samples’ weight was 13.5 ± 3.7 mg tissue per time point, and for the mouse samples 22.1 ± 2 mg tissue per time point. Next, 25 μl per mg tissue dry-ice cold extraction buffer (50% [v/v] methanol, 30% [v/v] acetonitrile and 20% [v/v] H_2_O, supplemented with [^13^C_4_]-succinate and valine-d_8_ as internal standards to a final concentration of 5 μM) was added to the Precellys tubes and the tissue was homogenized using a Precellys 24 tissue homogenizer (6500 rpm, 15 s; Bertin Instruments, France). The homogenizing program was run twice with 5 min interval on dry ice. Samples were then centrifuged at 17,000 rpm at 4 °C for 10 min and the supernatant was collected and incubated in a −20 °C freezer for 1 h. The centrifuging step was repeated two times and the supernatant was transferred to pre-chilled MS vials and were stored at −80 °C until analysis for metabolomics or absolute quantification of metabolites by liquid chromatography-tandem mass spectrometry (LC-MS/MS). For CSF samples, 3.5 μl of CSF was mixed with 100 μl of extraction buffer and internal standard, and the extraction was performed as above.

### 4.7 Metabolomics

Hydrophilic separation of metabolites was achieved using a Millipore Sequant ZIC-pHILIC analytical column (5 μm, 2.1 × 150 mm) equipped with a 2.1 × 20 mm guard column (both 5 mm particle size) with a binary solvent system. Solvent A was 20 mM ammonium carbonate, 0.05% ammonium hydroxide; Solvent B was acetonitrile. The column oven and autosampler tray were held at 40 °C and 4 °C, respectively. The chromatographic gradient was run at a flow rate of 0.200 mL/min as follows: 0–2 min: 80% B; 2–17 min: linear gradient from 80% B to 20% B; 17–17.1 min: linear gradient from 20% B to 80% B; 17.1–22.5 min: hold at 80% B. Samples were randomized and analyzed with LC–MS in a blinded manner with an injection volume was 5 μl. Pooled samples were generated from an equal mixture of all individual samples and analyzed interspersed at regular intervals within sample sequence as a quality control.

Metabolites were measured with a Thermo Scientific Q Exactive Hybrid Quadrupole-Orbitrap Mass spectrometer (HRMS) coupled to a Dionex Ultimate 3000 UHPLC. The mass spectrometer was operated in full-scan, polarity-switching mode, with the spray voltage set to +4.5 kV/-3.5 kV, the heated capillary held at 320 °C, and the auxiliary gas heater held at 280 °C. The sheath gas flow was set to 55 units, the auxiliary gas flow was set to 15 units, and the sweep gas flow was set to 0 unit. HRMS data acquisition was performed in a range of *m/z* = 70–900, with the resolution set at 70,000, the AGC target at 1 × 10^6^, and the maximum injection time (Max IT) at 120 ms. Metabolite identities were confirmed using two parameters [1]: precursor ion *m/z* was matched within 5 ppm of theoretical mass predicted by the chemical formula [2]; the retention time of metabolites was within 5% of the retention time of a purified standard run with the same chromatographic method. Chromatogram review and peak area integration were performed using the Tracefinder software (v 5.0, Thermo Fisher Scientific).

### 4.8 Metabolomics data analysis

The peak area for each detected metabolite was subjected to the “Filtering 80% Rule”, half minimum missing value imputation, and normalized against the total ion count (TIC). Samples were tested for outliers based on geometric distances of each point in the PCA score analysis as part of the muma package (v.1.4) [24]. Normalized LC-MS results were first explored by PCA using the R base package stats (v.4.2.0) (https://www.r-project.org/) with the function prcomp and visualized using the autoplot function of ggplot2 (v.3.3.6) [25] after loading the ggfortify package (v.0.4.14) [26]. Differential metabolite analysis comparing timepoint 0 versus timepoint X was performed on the mean values of the analytical repeats of the normalized data. In detail, the R package “gtools” (v.3.9.2) [27] was used to calculate the Log_2_FC using the functions “foldchange” and “foldchange2logratio” (parameter base = 2). The corresponding *p* value was calculated using the R base package stats (v.4.2.0) with the function “t.test” and adjusted using Benjamini Hochberg “BH”. Volcano plots were generated using the EnhancedVolcano package (v. 1.14.0) [28] and the metabolic pathways where assigned based on our a priori knowledge. Detailed code can be found under https://github.com/ChristinaSchmidt1/Succinate_in_ischemic_stroke.

### 4.9 LC-MS/MS quantification of succinate and malonate

To quantify succinate and malonate a LCMS-8060 mass spectrometer (Shimadzu, UK) with a Nexera X2 UHPLC system (Shimadzu, UK) was used as described before [29]. Briefly, metabolites were separated using a SeQuant® ZIC®-HILIC column (3.5 μm, 100 Å, 150 x 2.1 mm, 30 °C column temperature; MerckMillipore, UK) with a ZIC®-HILIC guard column (200 Å, 1 x 5 mm) at a flow rate of 200 μl/min with mobile phases of A) 10 mM ammonium bicarbonate and B) 100% acetonitrile. The MS was operated in negative ion mode with multiple reaction monitoring (MRM) and spectra acquired using Labsolutions software (Shimadzu, UK). Succinate and malonate were quantified using standard curves relative to [^13^C_4_]-succinate or [^13^C_3_]-malonate internal standard.

### 4.10 Mass spectrometry imaging

Mice underwent the tMCAO surgery as described and at the end of 45 min ischemia or 45 ischemia +5 min reperfusion mice were terminally anesthetized with pentobarbital and craniotomy was performed in order to rapidly dissect the whole brain and freeze in LN2-cooled isopentane. Tissues were embedded in a HPMC/PVP-based hydrogel following a previously published protocol [30] to create multi-tissue blocks. The blocks were cryo-sectioned to collect tissue sections of 10 μm thickness on a cryostat (Leica Biosystems, Germany). The sections were either thaw-mounted onto SuperFrost slides (Thermo Scientific, Germany) for DESI-MSI, ITO-coated slides for MALDI-MSI or TOMO Adhesion Microscope Slides (Matsunami Glass Ind. Ltd., Japan) for histological stains. Sections were collected at different tiers from approximately Bregma-3 mm to Bregma+5 mm with an interval spacing of 1 mm. A total of 8 tiers were collected and evaluated for the presence of infarct. A total of 6 tiers were analyzed to fully represent the metabolic heterogeneity within the tissues. The sections were air-dried and vacuum-packed for storage at −80 °C prior to MSI. DESI-MSI experiments were performed using a Q-Exactive plus mass spectrometer (Thermo Scientific, UK) with an automated DESI ion source (Prosolia Inc., USA). Data were acquired in negative ion mode between *m/z* 80 and 1000. The nominal mass resolution was set to 70,000. The injection time was 150 ms, resulting in a scan rate of 3.8 pixel/s. A home-built DESI sprayer was operated with a mixture of 95% methanol/5% water at 1.5 μl/min and nebulized with nitrogen at a back pressure of 6 bar. The spatial resolution was 100 μm. MALDI-MSI was performed on a RapifleX instrument (Bruker Daltonics, Bremen, Germany). 9-Aminoacridine (9-AA) was used as MALDI matrix. The mass spectrometer was operated in negative ion mode to collect data from *m/z* 100–900. The spatial resolution was set to 65 μm and a total of 500 laser shots were collected to generate the final spectra.

All data analysis was performed in the SCiLS lab software package (V. 2020b, Bruker Daltonics, Germany) where the brain tissue was delineated using a partial least squares machine learning classifier (PLS) based on manual annotations. The ischemic lesions were subsequently determined based on metabolic changes identified by PCA followed by bisecting k-Means clustering based on the most discriminant loadings of the relevant principal components. Sections collected for histological evaluation stained with hematoxylin and eosin (H&E) and imaged on an Aperio scanner (Leica Biosystems, Germany) at 20 × resolution.

### 4.11 Statistics

For comparing multiple groups one-way analysis of variance (ANOVA) was used followed by Tukey's test. For infarct size comparisons, non-parametric Kruskal-Wallis test was performed with Dunn's *post-hoc* for multiple comparisons. Data are presented as mean values ± SD or SEM and *p* value less than 0.05 is considered significant. All statistical analysis was carried out using Graphpad Prism version 7.0e.

### 4.12 Study approval

Ethical approval for the analysis of the brain samples in the human study was obtained from NRES Committee East of England - Cambridge South (REC Reference 18/EE/0172, Biorepository number A094613). All animal procedures were carried out in accordance with the UK Animals (Scientific Procedures) Act 1986 and the University of Cambridge Animal Welfare Policy. Procedures were approved to be carried out under the Project License PP4344323.

## Author contributions

AM, MPM and TK designed the study and wrote the manuscript. AM designed and performed the experiments and analyzed the data. HAP contributed to experimental design and performed the mass spectrometric analysis for succinate and malonate. AD and RG performed and analyzed the imaging mass spectrometry data. RM contributed to human sample experimental design and provided the human samples with MH's assistance. CS, MY and CF performed the metabolomics and analyzed the data. ASA, JL, NB, SP and LPJ contributed to animal experiments. All authors read, edited, and revised the manuscript.

## Declaration of competing interest

HAP, MPM and TK have submitted patent applications in the therapeutic use of malonate in treating IR injury.

## Data Availability

Data will be made available on request.

## References

[1] Feigin V.L., Stark B.A., Johnson C.O., Roth G.A., Bisignano C., Abady G.G. (2021). Global, regional, and national burden of stroke and its risk factors, 1990–2019: a systematic analysis for the Global Burden of Disease Study 2019. Lancet Neurol..

[2] Jovin T.G., Nogueira R.G., Lansberg M.G., Demchuk A.M., Martins S.O., Mocco J. (2022). Thrombectomy for anterior circulation stroke beyond 6 h from time last known well (AURORA): a systematic review and individual patient data meta-analysis. Lancet.

[3] Albers G.W., Marks M.P., Kemp S., Christensen S., Tsai J.P., Ortega-Gutierrez S. (2018). Thrombectomy for stroke at 6 to 16 hours with selection by perfusion imaging. N. Engl. J. Med..

[4] Lansberg M.G., Straka M., Kemp S., Mlynash M., Wechsler L.R., Jovin T.G. (2012). MRI profile and response to endovascular reperfusion after stroke (DEFUSE 2): a prospective cohort study. Lancet Neurol..

[5] Nogueira R.G., Jadhav A.P., Haussen D.C., Bonafe A., Budzik R.F., Bhuva P. (2018). Thrombectomy 6 to 24 hours after stroke with a mismatch between deficit and infarct. N. Engl. J. Med..

[6] Abou-Chebl A. (2010). Endovascular treatment of acute ischemic stroke may be safely performed with no time window limit in appropriately selected patients. Stroke.

[7] Mokin M., Ansari S.A., McTaggart R.A., Bulsara K.R., Goyal M., Chen M. (2019). Indications for thrombectomy in acute ischemic stroke from emergent large vessel occlusion (ELVO): report of the SNIS Standards and Guidelines Committee. J. NeuroIntervent. Surg..

[8] Mizuma A., You J.S., Yenari M.A. (2018). Targeting reperfusion injury in the age of mechanical thrombectomy. Stroke.

[9] Slegtenhorst B.R., Dor F.J., Rodriguez H., Voskuil F.J., Tullius S.G. (2014). Ischemia/reperfusion injury and its consequences on immunity and inflammation. Curr Transplant Rep.

[10] Chouchani E.T., Pell V.R., Gaude E., Aksentijević D., Sundier S.Y., Robb E.L. (2014). Ischaemic accumulation of succinate controls reperfusion injury through mitochondrial ROS. Nature.

[11] Yin Z., Burger N., Kula-Alwar D., Aksentijević D., Bridges H.R., Prag H.A. (2021). Structural basis for a complex I mutation that blocks pathological ROS production. Nat. Commun..

[12] Chouchani E.T., Methner C., Nadtochiy S.M., Logan A., Pell V.R., Ding S. (2013). Cardioprotection by S-nitrosation of a cysteine switch on mitochondrial complex I. Nat. Med..

[13] Valls-Lacalle L., Barba I., Miró-Casas E., Ruiz-Meana M., Rodríguez-Sinovas A., García-Dorado D. (2018). Selective inhibition of succinate dehydrogenase in reperfused myocardium with intracoronary malonate reduces infarct size. Sci. Rep..

[14] Valls-Lacalle L., Barba I., Miró-Casas E., Alburquerque-Béjar J.J., Ruiz-Meana M., Fuertes-Agudo M. (2016). Succinate dehydrogenase inhibition with malonate during reperfusion reduces infarct size by preventing mitochondrial permeability transition. Cardiovasc. Res..

[15] Prag H.A., Aksentijevic D., Dannhorn A., Giles A.V., Mulvey J.F., Sauchanka O. (2022). Ischemia-selective cardioprotection by malonate for ischemia/reperfusion injury. Circ. Res..

[16] Sutherland B.A., Neuhaus A.A., Couch Y., Balami J.S., DeLuca G.C., Hadley G. (2016). The transient intraluminal filament middle cerebral artery occlusion model as a model of endovascular thrombectomy in stroke. J. Cerebr. Blood Flow Metabol..

[17] Martin J.L., Costa A.S.H., Gruszczyk A.V., Beach T.E., Allen F.M., Prag H.A. (2019). Succinate accumulation drives ischaemia-reperfusion injury during organ transplantation. Nat Metab.

[18] Li Y., Wang T., Zhang T., Lin Z., Li Y., Guo R. (2020). Fast high-resolution metabolic imaging of acute stroke with 3D magnetic resonance spectroscopy. Brain.

[19] Zhu H., Barker P.B. (2011). MR spectroscopy and spectroscopic imaging of the brain. Methods Mol. Biol..

[20] Ganesana M., Venton B.J. (2018). Early changes in transient adenosine during cerebral ischemia and reperfusion injury. PLoS One.

[21] Rudolphi K.A., Schubert P., Parkinson F.E., Fredholm B.B. (1992). Adenosine and brain ischemia. Cerebrovasc. Brain Metab. Rev..

[22] Williams-Karnesky R.L., Stenzel-Poore M.P. (2009). Adenosine and stroke: maximizing the therapeutic potential of adenosine as a prophylactic and acute neuroprotectant. Curr. Neuropharmacol..

[23] Swanson R.A., Morton M.T., Tsao-Wu G., Savalos R.A., Davidson C., Sharp F.R. (1990). A semiautomated method for measuring brain infarct volume. J. Cerebr. Blood Flow Metabol..

[24] Gaude E., Chignola F., Spiliotopoulos D., Spitaleri A., Ghitti M., Garcia-Manteiga J M. (2013). Muma, an R package for metabolomics univariate and multivariate statistical analysis. Current Metabolomics.

[25] Wickham H. (2016).

[26] Tang Y., Horikoshi M., Li W. (2016). ggfortify: unified interface to visualize statistical results of popular R packages. Rev. Javer..

[27] Warnes G.R., Bolker B., Lumley T. (2015). https://cran.r-project.org/web/packages/gtools/index.html.

[28] Blighe K., Rana S., Turkes E., Ostendorf B., Grioni A., EnhancedVolcano M.L. (2020).

[29] Prag H.A., Pala L., Kula-Alwar D., Mulvey J.F., Luping D., Beach T.E. (2022). Ester prodrugs of malonate with enhanced intracellular delivery protect against cardiac ischemia-reperfusion injury in vivo. Cardiovasc. Drugs Ther..

[30] Dannhorn A., Kazanc E., Ling S., Nikula C., Karali E., Serra M.P. (2020). Universal sample preparation unlocking multimodal molecular tissue imaging. Anal. Chem..

