# Supplementary material for: Targeting succinate metabolism to decrease brain injury upon mechanical thrombectomy treatment of ischemic stroke
Source: Redox Biol. 2023 Jan 2;59:102600. doi: 10.1016/j.redox.2023.102600 (PMC9841348; doi:10.1016/j.redox.2023.102600)
Supplement: Multimedia component 1 [file mmc1.pdf]

Supplemental Table 1. Characteristics of human brain samples.

Patient number	Age	Sex	Tumor type	Anatomical location	Ischemia time points (min)	Time to freeze sample (s)
1	54	Female	Glioblastoma	Frontal	0,15,60,120	~ 60
2	67	Male	Oligodendroglioma	Parietal	0,5,15,60,120	~ 60
3	49	Male	Glioblastoma	Occipital	0,5,15,60,120	~ 60
4	71	Female	Glioblastoma	Parietal	0,5,15,60,120	68
5	65	Female	Metastatic	Temporal	0,15,60	112
6	70	Male	Glioblastoma	Frontal	0,15,60	32
7	65	Female	Metastatic	Cerebellar	0,15,60	16
8	44	Male	Glioblastoma	Temporal	0,5,15,60	36

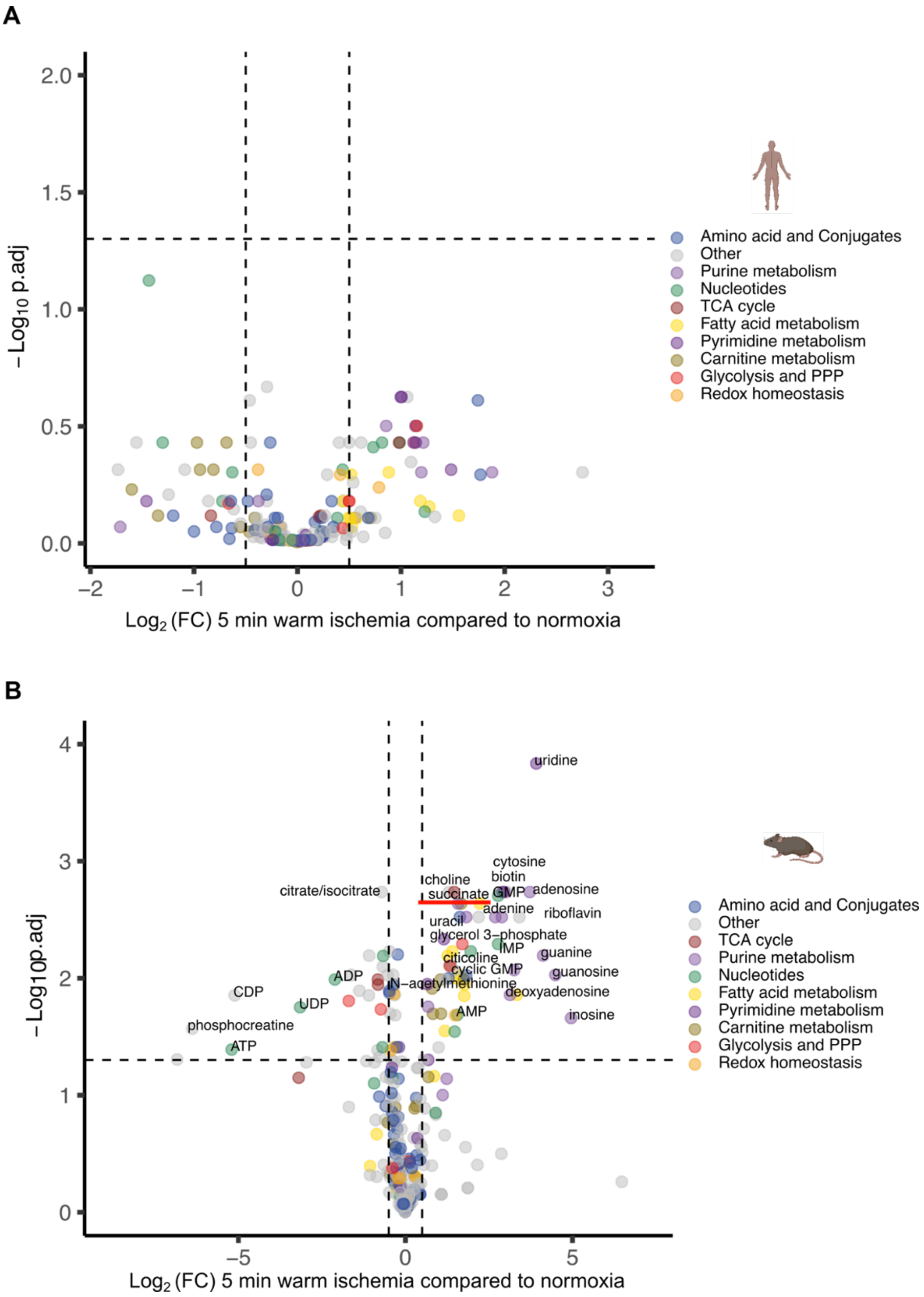

Supplemental Figure 1. **Volcano plots showing changes in metabolite levels following 5 min *ex vivo* warm ischemia compared to normoxia**. Human **(A)** and mouse **(B)** brain tissue data are shown. The volcano plots show -Log_{10}(adjusted p value (p.adj.) calculated by the p-value using the Benjamini-Hochberg procedure), plotted against Log_2(fold change (FC)), where fold change is that comparing the change in abundance of metabolites after 5 min warm ischemia compared to normoxia. Metabolite abundances are given in Supplemental Tables 2 & 3 and Log_2(FC) and -Log_{10}(p.adj.) were calculated from these data. Each dot represents the mean of 4-8 replicates for human and of 4 replicates for mouse. Metabolites from different metabolic pathways are color-coded and key metabolites and/or those that change to the greatest extent are labeled.

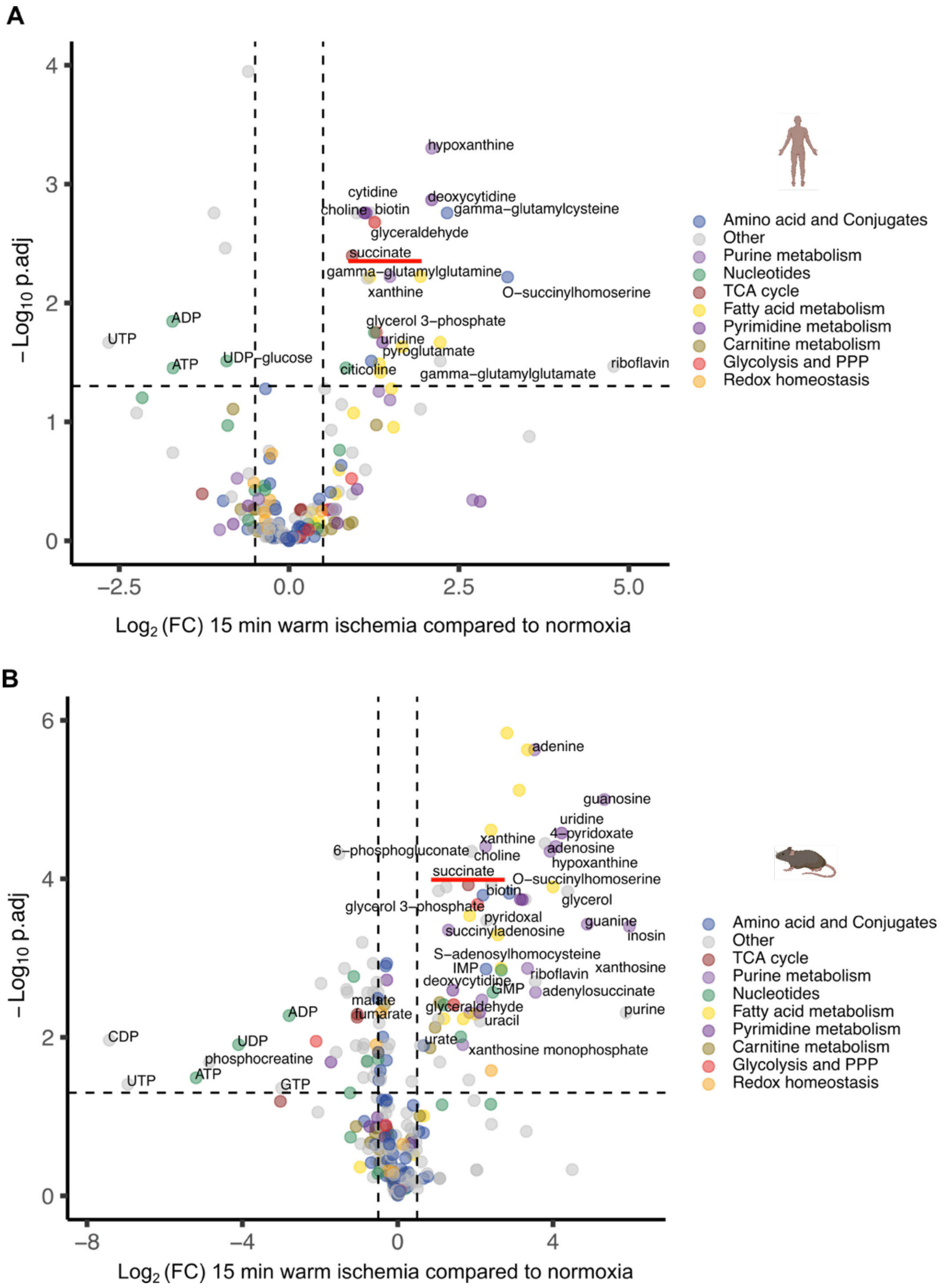

Supplemental Figure 2. **Volcano plots showing changes in metabolite levels following 15 min *ex vivo* warm ischemia compared to normoxia**. Human **(A)** and mouse **(B)** brain tissue data are shown. The volcano plots show -Log_{10}(adjusted P value (p.adj.) show -Log_{10}(adjusted p value (p.adj.) calculated by adjusting the p-value using the Benjamini-Hochberg procedure), plotted against Log_2(fold change (FC)), where fold change is that comparing the change in abundance of metabolites after 15 min warm ischemia compared to normoxia. Metabolite abundances are given in Supplemental Tables 2 & 3 and Log_2(FC) and -Log_{10}(P.adj.) were calculated from these data. Each dot represents the mean of 8 replicates for human and of 4 replicates for mouse. Metabolites from different metabolic pathways are color-coded and key metabolites and/or those that change to the greatest extent are labeled

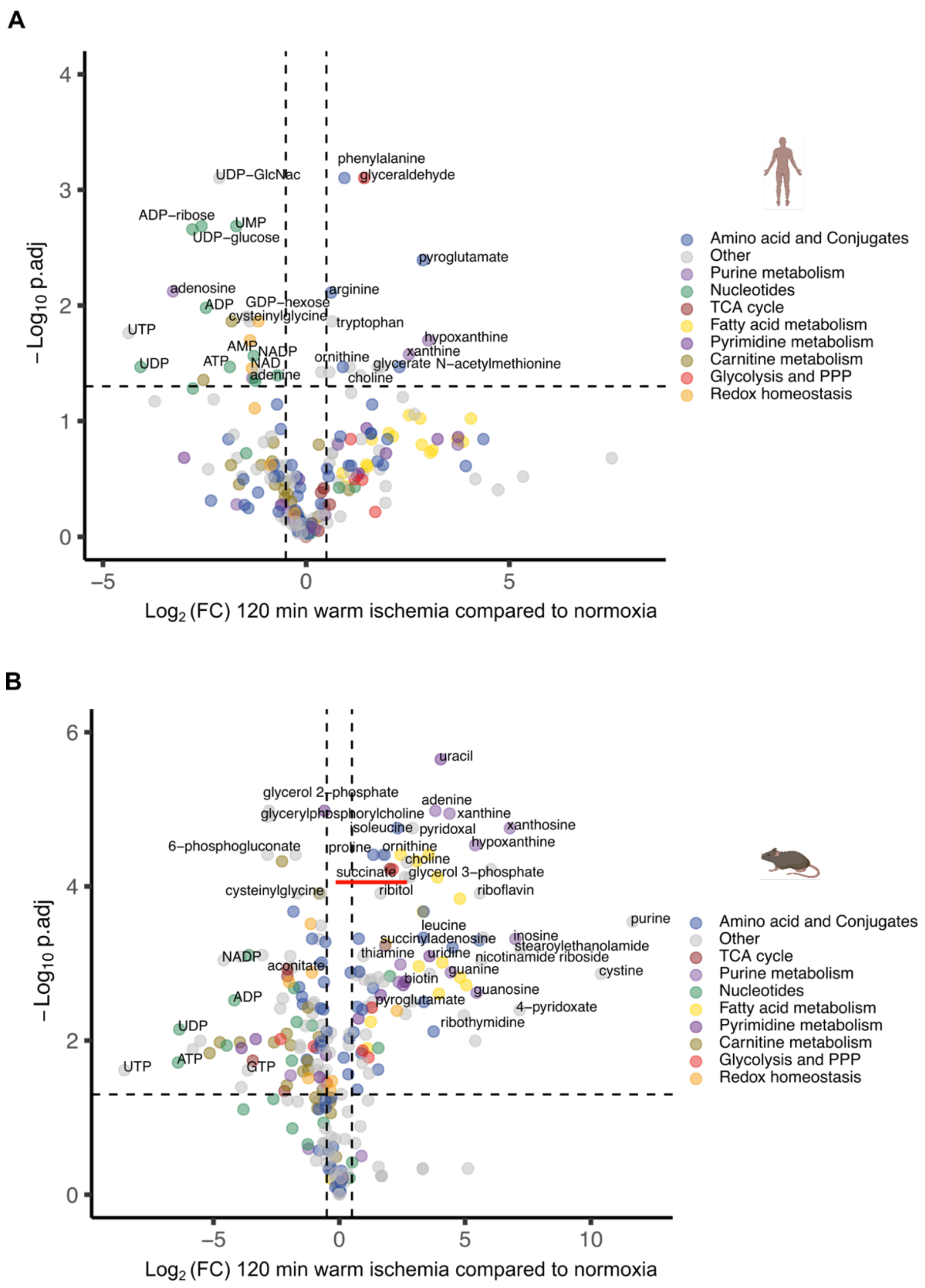

Supplemental Figure 3. **Volcano plots showing changes in metabolite levels following 120 min *ex vivo* warm ischemia compared to normoxia**. Human **(A)** and mouse **(B)** brain tissue data are shown. The volcano plots show $-Log_{10}$(adjusted p value (p.adj.) show $-Log_{10}$(adjusted p value (p.adj.) calculated by adjusting the p-value using the Benjamini-Hochberg procedure), plotted against Log_2(fold change (FC)), where fold change is that comparing the change in abundance of metabolites after 120 min warm ischemia compared to normoxia. Metabolite abundances are given in Supplemental Tables 2 & 3 and Log_2(FC) and $-Log_{10}$(p.adj.) were calculated from these data. Each dot represents the mean of 4-8 replicates for human and of 4 replicates for mouse. Metabolites from different metabolic pathways are color-coded and key metabolites and/or those that change to the greatest extent are labeled.

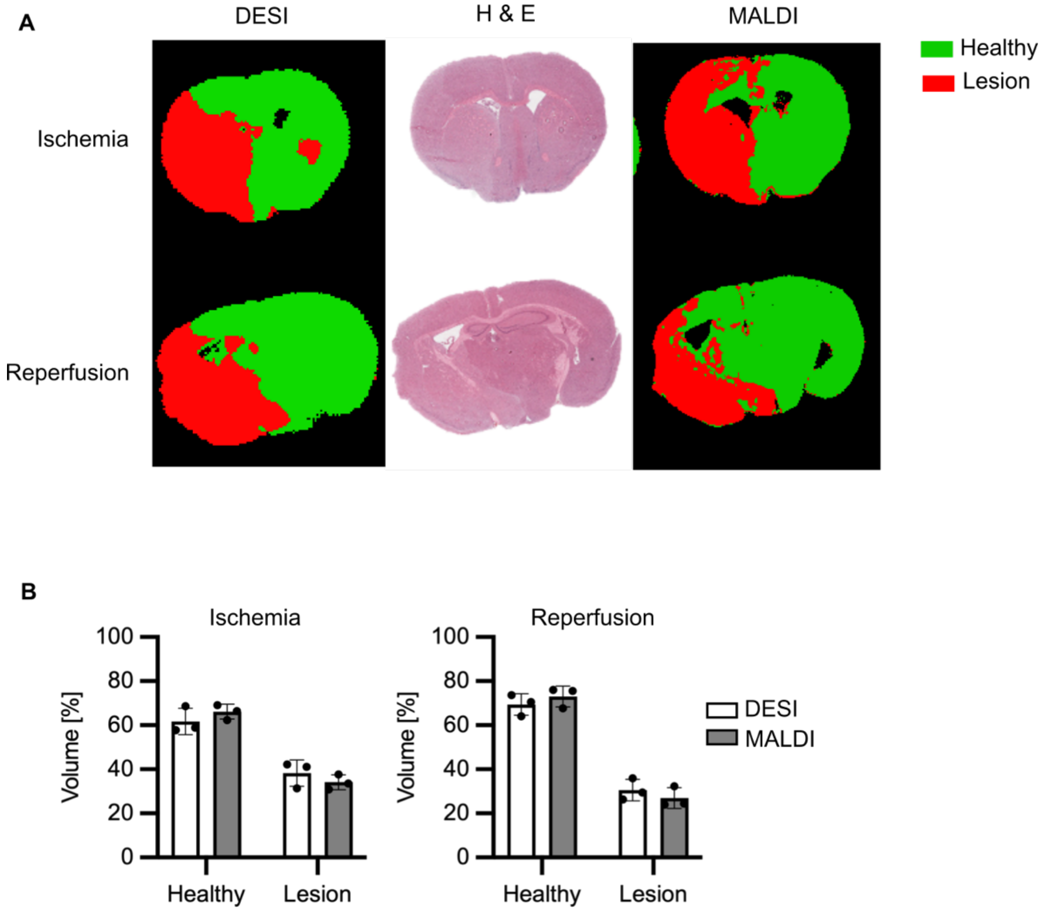

Supplemental Figure 4. Agreement between DESI and MALDI image segmentation analysis. **(A)** Representative mass spectrometry and histology (H & E) images and the segmentations by principal component analysis. Brain samples obtained from mice exposed to 45 min of tMCAO (ischemia) or 45 min tMCAO and 5 min of reperfusion (reperfusion). (**B**) Volumetric analysis of lesion and healthy tissue from sections imaged and segmented after DESI and MALDI mass spectrometry imaging method. Volumes are presented as percentage of total volume. n = 3. Data are presented as mean ± SD. Comparisons was performed by two-way ANOVA and Sidak's test and no significant differences were observed between DESI and MALDI segmentation.

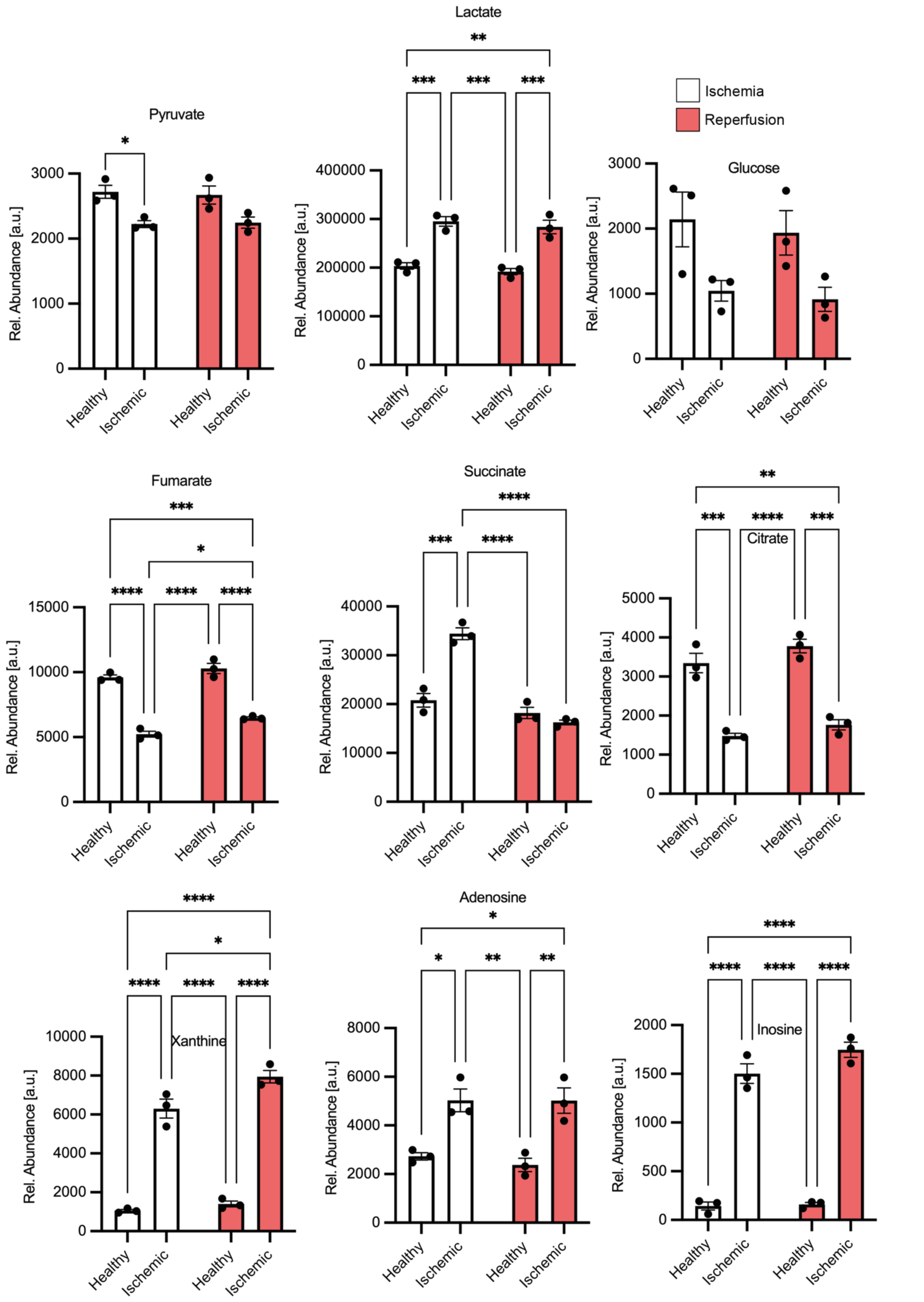

Supplemental Figure 5. Distribution of metabolites in healthy and ischemic tissue after ischemia and reperfusion. Quantification of key metabolites in glycolysis, Krebs cycle and purine metabolism pathways after ischemia and reperfusion by mass spectrometry imaging.

Comparisons between groups was performed using two-way ANOVA and Sidak's test, n = 3. Data are presented as mean ± SEM.

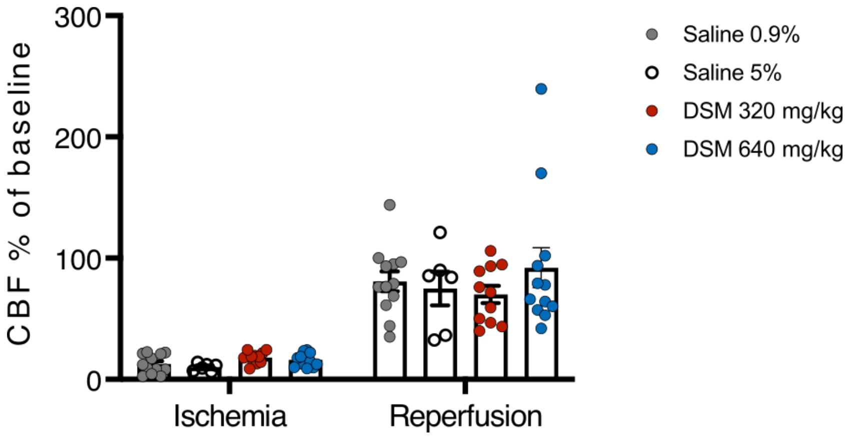

Supplemental Figure 6. Laser doppler flowmetry of cerebral blood flow (CBF) during tMCAO. Mice were exposed to tMCAO for 45 min of ischemia and then either saline or disodium malonate (DSM) was given intravenously starting 10 min before reperfusion until 10 min after reperfusion. Laser doppler flowmetry of cerebral blood flow (CBF) showed the extent of ischemia and reperfusion, but there was no significant difference observed among treatment groups (n = 6-12, 2-way ANOVA and Tukey's post hoc test.). Data are presented as mean ± SEM.
